# Much Ado About Gender

**DOI:** 10.1007/s10508-025-03330-z

**Published:** 2025-11-23

**Authors:** Tove Solander

**Affiliations:** School of Humanities, Education and Social Sciences, Örebro University, 701 82 Örebro, Sweden

## How Many Sexes Are There?

Two---in humans, other animals, and most other species with sexual reproduction. The fact that not all individual organisms can be neatly sexed does not change this number (see Moi, 1999, pp. 39–40; Hull, 2006, pp. 54–74). There are life forms like fungi with more “mating types,” but never more than two involved in reproduction at the same time. Two is enough to introduce genetic variation. Grosz, a feminist philosopher with an unusual interest in evolutionary theory, opens for the possibility that there may in future be more---but not fewer---than two: “The human, as Darwin made clear, is the result of and an elaboration on sexual difference, and thus the human, and indeed the beyond-the-human necessarily take on (at least) two forms” (Grosz, 2005, p. 42). This is because evolution does not reverse into less differentiated forms: “Sexual difference, once introduced as a modality of biological survival, is unlikely to be removed, only complexified, elaborated, developed further, perhaps even beyond the human” (Grosz, 2004, p. 67). Nobody knows what evolution might bring, but in my view this post-human future is less relevant to us at present. The answer has been two for a very large part of life on earth for a very long time.

## How Many Genders Are There?

That depends on how you define “gender.” When used as a euphemism for “sex,” naturally, two. Leaving this non-definition aside, I shall give my best answers according to definitions drawing on---while often contesting---the sex/gender distinction.

For “gender identity” in the original formulation of either a sense of which sex one belongs to or an identification with the associated “gender role” (the early sexologists coining the term already mix these things up), also two (see Hausman, 1995, pp. 102–109). More recent formulations struggle to accommodate as many as there are individuals claiming a unique gender, which leads to circular definitions and raises the question if “gender” really is the best word for idiosyncratic identities. Gender is also used synonymously with “sex role” or “gender role.” There are psychological, sociological, and feminist variations on this, but since it is about social roles assigned based on sex, the answer is again two.

As for feminist theories of gender, many of them analyze “gender systems” on a structural level. In these gender-system theories, the answer is usually two: man/masculine and woman/feminine, hierarchically arranged and defined in opposition to one another rather than with reference to sex differences (see Scott, 1986). In an early, important elaboration of gender theory, Rubin (1975) writes: “Gender is a socially imposed division of the sexes” (p. 179). However, Rubin still refers to “males” and “females,” indicating that her sex/gender system is based on an understanding of two sexes and two (corresponding) genders. In MacKinnon’s (1982) radical feminist version of the gender system, “man” and “woman” are social roles depending mainly on your position in sexual intercourse. Drawing this logic to the extreme, MacKinnon should agree with Wittig (1980) that “lesbians are not women,” while presumably homosexual male bottoms are. In other words, there are still only two genders, although they do not align perfectly with sex. The liberal feminist Okin (1989) defines gender as “*the deeply entrenched institutionalization of sexual difference*” [italics in original] (p. 6), which must be removed in order to achieve a just society. In other variations on the feminist gender-system theme, Young (1994) describes gender as a “social collective,” Haslanger (2000) as a “social position,” and Risman (2004) as a “social structure.”

Although Haslanger leaves open the possibility of non-hierarchical and multiple genders, the answer tends to be two in feminist gender-system theories because “gender” is used as a tool of critical analysis rather than, say, utopian thinking. The idea is to abolish gender---and even “women” and “men” when conceived of as “genders.” These feminists, who can be described as egalitarian whether of a liberal, Marxist or radical variety, tend to view any division between the sexes as automatically entailing gender inequality. They therefore get stuck on the equality-versus-difference paradox and struggle with accommodating issues such as childbirth. Firestone (2015) suggested the radical solution of getting rid of it entirely and making reproduction gender neutral. Although few go so far, sex differences are often dismissed as insignificant, or as so heavily overlaid with cultural meanings as to be unknowable.

In other words, sex fades from view within academic feminism, leaving only the gender half of the dichotomy. The sex/gender distinction has been debated ever since the concept of gender was introduced into feminist theory, among other things for upholding a Cartesian mind/body split and for “naturalizing” sex. Just to take a few examples, Friedman (1991) defends the distinction as analytically useful even if both sex and gender are ultimately socially constructed. Nicholson (1994) disagrees and berates even those feminists who use only “gender” for implicitly grounding it in sex. My impression is that the current consensus within gender studies is that “gender” denotes an understanding of sex as culturally constructed, rather than of sex and gender as distinct in any meaningful sense.

West and Zimmerman’s (1987) theory of “doing gender” can be characterized as an elaboration on gender-system theories and a predecessor to Butler’s “gender performativity.” Again the answer is two, as West and Zimmerman view the doing of gender as a cultural imperative upholding the sex categories of “man” and “woman” and as more fundamental than other social roles, which are all gendered. Butler (1999) combines this idea with Rubin’s notion that compulsory heterosexuality is at the heart of the gender system and produces gender difference. However, Butler’s “heterosexual matrix” seems to oscillate between a strict gender binary and almost infinite possibilities for doing gender differently. On the one hand, all “genders” but masculine males desiring women and feminine females desiring men are described as unintelligible; on the other, Butler’s three variables sex--gender--sexuality theoretically enable at least eight different “genders.” In a characteristic string of rhetorical questions, Butler (1999) writes that when gender is theorized as “a free-floating artifice” with no mimetic relation to sex, “there is no reason to assume that genders ought also to remain as two” (p. 10). In a complicated theoretical move, Butler also flips the sex/gender dichotomy, insisting that gender precedes and produces sex.

Hull (2006, pp. 83–111) demonstrates the fallacy of poststructuralist gender theories such as Butler’s. Biological sex is employed in two contradictory ways: as “really” infinitely more complex than just male or female and as a mere construct of scientific discourse. On the one hand, there is frequent rhetorical pointing to exceptions calling the two-sex norm into question, as though reality mattered. On the other hand, the sex/gender distinction is rejected (or deconstructed) and both terms are ultimately understood as discursive constructs. Typically, some version of the “gender binary” is held up as what produces the (illusion of) two sexes. But if “sex” is a constructed category imposed on a chaotic or unknowable reality, it is no more or less than we make it out to be. If the theory is that binary culture makes it binary, so it is. Ultimately, then, sex is binary because gender is and gender is binary because sex is. In my view, such hyper-constructivist, free-floating gender-system theories have no more explanatory value or critical potential than speculations about living in the Matrix. Furthermore, they are anthropocentric, since we are just one sexually reproducing animal among many.

A common way of defining gender, including in feminist theory, is by reference to masculinity and femininity. For instance, Friedman (1991) gives the definition “the socio-cultural constructs of femininity and masculinity” (p. 200). A popular conceptualization of gender as femininity and masculinity views it as something like a scale with Barbie and G. I. Joe at the opposite ends and most, if not all, actual human beings somewhere in between. A variation of this is the Bem Sex-Role Inventory, which puts masculinity and femininity on separate scales rather than opposite ends and describes a high score on both as desirable “psychological androgyny” (Bem, 1974). On models like this, the genders are either still two---seen as ideal types of masculinity and femininity no-one lives up to---or infinite individual variations, each their own “gender.” The latter approaches popular descriptions of infinite “gender identities” and “gender expressions” and raises similar objections.

Depending on whether you think sex matters, gender defined in binary terms as masculinity/femininity might enable four different “genders.” Halberstam (1998) insists that female masculinity is distinct from and not derivative of male masculinity. As suggested by the examples of Halberstam, Butler, and Wittig, those who wish to argue for more than two genders tend to turn either to sexual minorities (queer studies teem with examples such as inverts, butches and queens) or to so-called “third-gender cultures,” in which there are additional social categories distinct from “men” and “women.” Usually, the additional gender is sex-specific and reserved for feminine and/or androphilic males. There is no necessary symmetry in designating a corresponding gender category for masculine and/or gynephilic females*–*or excusing an equal share of women from heterosexual reproduction. Masculine women tend to achieve cultural recognition as singular individuals rather than as a social group, and often conditioned on being “virgins” (although whether sex between women counts is a different matter).

These examples suggest that even when accounting for the possibility of more than two genders, gender retains some connection to sex, in both senses of the word. Therefore, I am partial to a critical--realist feminist understanding of sex as the basis of gender (see Hull, 2006, pp. 104–105). According to this logic, the genders are (usually) two because the sexes (always) are. A critical--realist view might allow for more than two genders, but not endless numbers. First of all, gender needs to retain a grounding in sex in order to be meaningfully called gender. Robust arguments have to be made for proclaiming a new gender, such as it being socially recognized in the real world and applied to a reasonably distinct group. For instance, if widows in a certain society have a markedly different social position than other women, it could be considered a separate gender, while *tumblr* genders do not count.

Whether there might be more than two genders also depends on what your gender concept is supposed to do. If it is used to describe culturally variable gender systems, it makes sense to argue for more than two genders. If it is used as a tool of critical analysis, however, it makes no sense. Unless you think like a true poststructuralist that you can dismantle the master’s house using the master’s tools, to paraphrase Lorde (1984). I agree with Hausman (1995, p. 197) and Moi (1999, p. 29) that attempting to vanish the gender system by multiplying genders is a futile feminist strategy.

Finally, the answer could also be fewer than two, as exemplified by de Beauvoir. According to Moi (1999), de Beauvoir has been falsely accused of inventing the feminist sex/gender distinction due to an Anglophone misreading of her existentialist feminism (pp. 72–79). When de Beauvoir says “woman” she does not mean a “gender” but the body as situation and lived experience. She does, however, also have a concept close to “gender norms” which Moi (1999) translates as “myths of femininity” (pp. 80–81). In this sense of “gender,” there is only one for de Beauvoir. Man is the unmarked, un-gendered universal while Woman is his marked, gendered Other. Drawing on de Beauvoir, Wittig (1983) states this outright:

Gender is used here in the singular because indeed there are not two genders. There is only one: the feminine, the “masculine” not being a gender. For the masculine is not the masculine but the general. (p. 64)

Wittig’s, as well as de Beauvoir’s, feminist project is to free women from “gender” (the male projection of otherness) and grant them the status of universal human.

In a variation on this theme, sexual difference feminists such as Irigaray might answer: one, the masculine. According to Irigaray, androcentric culture does not recognize sexual difference but only Man and his projected mirror-image Woman. What sexual difference feminists refer to as “the feminine” has not yet found expression and we do not know what culturally recognizing it might entail. However, it is inexact to translate this into the language of gender. Rather, sexual difference feminists do not particularly care about “gender” since it exists entirely within the androcentric or phallogocentric order anyway (see Butler, 1999, pp. 14–18; Grosz, 2005, pp. 173–177).

Gatens (1995) compares the over-emphasis on gender to treating only the symptom and ignoring the root cause, as though the connection between sex and gender was arbitrary. She writes: “If one accepts the notion of the sexually specific subject, that is, the male or female subject, then one must dismiss the notion that patriarchy can be characterized as a system of social organization that valorizes the masculine *gender* [italics in original] over the feminine gender. Gender is not the issue; sexual difference is” (Gatens, 1995, p. 9). I agree. Gender is certainly culturally significant and worth doing research about. It can even be sexy. But from a feminist perspective, I increasingly feel that the focus on gender---to the detriment of sex---is a *cul-de-sac*.

## Feminism Beyond Gender?

Feminists and other critical theorists have long criticized the sexological concept of gender for medicalizing, commodifying, and depoliticizing what might otherwise be analyzed as “sex-role oppression” (Raymond, 1994, p. 9) or “sex-based stereotypes” (Moi, 1999, p. 82). A similar critique could be directed at the feminist concept of gender, which has become self-perpetuating within “gender studies” and so on. Moi’s introduction to her 1999 essay “What is a woman?” is still highly relevant:

In contemporary feminist theory so much energy is spent keeping the spectre of biologically based essentialism at bay that it is easy to forget that generalizations about gender may be just as oppressive as generalizations about sex. In many situations today biological determinism is not the most pressing obstacle to an emancipatory understanding of what a woman is. (p. 7)

I feel that academic feminists in Butler’s footsteps have been too busy beating the zombified cadaver of biologism to notice how reactionaries and anti-feminists love—rather than “are afraid of”—gender. Grosz (2005) suggested twenty years ago that “it is time to move beyond the very language of identity and gender, to look at other issues left untouched, questions unasked, and assumptions unelaborated” (p. 171). I think it is now high time indeed.

Grosz (2004, p. 259; 2005, p. 168; 2011, p. 148) puts sexual difference on par with mortality as one of humanity’s eternal questions, a question necessitating feminism as one of several possible answers. Feminism cannot (dis)solve sexual difference once and for all, as the various gender-abolitionist strands of feminism hope for (Grosz, 2005, p. 162). Sexual difference will remain for at least as long as the human remains human. Furthermore, unlike versions of feminism in which an oppressed group demands freedom and recognition from an oppressor (Grosz, 2005, p. 167), women are already in some important sense free and autonomous, else feminism would not be possible (Grosz, 2011, p. 73). As a feminist and a female human, I much rather inhabit Grosz’ queerly differentiated evolutionary universe than Butler’s claustrophobically anthropocentric linguistic labyrinth.
